# Poor Decision Making Is a Consequence of Cognitive Decline among Older Persons without Alzheimer’s Disease or Mild Cognitive Impairment

**DOI:** 10.1371/journal.pone.0043647

**Published:** 2012-08-20

**Authors:** Patricia A. Boyle, Lei Yu, Robert S. Wilson, Keith Gamble, Aron S. Buchman, David A. Bennett

**Affiliations:** 1 Rush Alzheimer’s Disease Center, Rush University Medical Center, Chicago, Illinois, United States of America; 2 Department of Behavioral Sciences, Rush University Medical Center, Chicago, Illinois, United States of America; 3 Department of Neurological Sciences, Rush University Medical Center, Chicago, Illinois, United States of America; 4 Department of Finance, DePaul University, Chicago, Illinois, United States of America; Oregon Health & Science University, United States of America

## Abstract

**Objective:**

Decision making is an important determinant of health and well-being across the lifespan but is critical in aging, when many influential decisions are made just as cognitive function declines. Increasing evidence suggests that older adults, even those without dementia, often make poor decisions and are selectively vulnerable to scams. To date, however, the factors associated with poor decision making in old age are unknown. The objective of this study was to test the hypothesis that poor decision making is a consequence of cognitive decline among older persons without Alzheimer’s disease or mild cognitive impairment.

**Methods:**

Participants were 420 non-demented persons from the Memory and Aging Project, a longitudinal, clinical-pathologic cohort study of aging in the Chicago metropolitan area. All underwent repeated cognitive evaluations and subsequently completed assessments of decision making and susceptibility to scams. Decision making was measured using 12 items from a previously established performance-based measure and a self-report measure of susceptibility to scams.

**Results:**

Cognitive function data were collected over an average of 5.5 years prior to the decision making assessment. Regression analyses were used to examine whether the prior rate of cognitive decline predicted the level of decision making and susceptibility to scams; analyses controlled for age, sex, education, and starting level of cognition. Among 420 persons without dementia, more rapid cognitive decline predicted poorer decision making and increased susceptibility to scams (p’s<0.001). Further, the relations between cognitive decline, decision making and scams persisted in analyses restricted to persons without any cognitive impairment (i.e., no dementia or even mild cognitive impairment).

**Conclusions:**

Poor decision making is a consequence of cognitive decline among older persons without Alzheimer’s disease or mild cognitive impairment, those widely considered “cognitively healthy.” These findings suggest that even very subtle age-related changes in cognition have detrimental effects on judgment.

## Introduction

The recent reconceptualization of Alzheimer’s disease (AD) reflects increased awareness that the pathophysiologic changes of AD develop slowly over many years, even decades, before cognitive impairment becomes clinically evident [1], [2]. Further, accumulating AD pathology leads to cognitive decline that precedes the clinical diagnosis of AD and even its precursor, mild cognitive impairment (MCI) [1], [2]. Older persons who exhibit cognitive decline but do not yet meet accepted criteria for MCI or AD may be most likely to benefit from early intervention and offer a unique opportunity to reduce the public health burden posed by AD [2]. Thus, there is an urgent need to clarify the consequences of cognitive decline among persons without AD or MCI in order to identify those who may benefit most from early intervention. To date, however, the consequences of cognitive decline among persons without MCI or AD remain unknown.

In this study, we tested the hypothesis that poor decision making is a consequence of cognitive decline among older persons without AD or MCI. Decision making is a complex behavior that requires higher order cognitive functions and is critical in aging, when some of life’s most influential decisions are made (e.g., intergenerational transfers of wealth, end of life healthcare decisions). For reasons unknown, older persons are highly vulnerable to poor decision making and frequently make suboptimal financial and healthcare choices (e.g., take social security distributions early, fail to enroll in Medicare part D) [3]–[5]. Further, older persons comprise the vast majority of fraud victims and are deprived of a substantial proportion of the more than 100 billion dollars lost annually to financial and health-related scams in the United States [6]–[7]. Although studies have shown that aspects of decision making are impaired among persons with dementia and MCI, these persons do not account for the widespread susceptibility of older persons to poor decision making and scams [8]–[10]. To date, however, virtually nothing is known about the factors associated with poor decision making among persons without dementia or MCI, the group generally considered “cognitively healthy.”

Data are from the Memory and Aging Project, a longitudinal cohort study of aging that began in 1997 and is ongoing; beginning in 2010, a decision making study was added [11]. Using data from 420 non-demented persons, we first tested the hypothesis that the rate of cognitive decline prior to the decision making assessment predicted the level of decision making. Then, we excluded persons with MCI at the time of decision making assessment and directly examined the relation of the prior rate of cognitive decline with decision making among persons without any overt cognitive impairment (i.e., no dementia or MCI). Finally, we repeated these analyses with a measure of susceptibility to scams as an alternate decision making outcome.

## Methods

### Participants

Participants were from the Memory and Aging Project, an ongoing longitudinal study of chronic conditions of aging and are residents of approximately 40 senior housing facilities in the Chicago metropolitan area. Participation involves risk factor assessment, detailed annual clinical evaluations including medical history, neurological and neuropsychological examinations, and organ donation at death. The study was approved by the Institutional Review Board of Rush University Medical Center, and written informed consent was obtained following a detailed, in person presentation of the risks and benefits associated with participation. All potential participants who declined to participate or otherwise did not participate were not disadvantaged in any way by not participating in the study.

The Memory and Aging Project began in 1997 and enrollment is ongoing. The decision making assessment began in 2010. At the time of these analyses, 1543 participants had completed the baseline evaluation for the parent study; of those, 429 died before the decision making project began, 96 refused further participation, and clinical diagnoses were pending in 26. Of the remaining 992 potentially eligible persons, 620 completed decision making assessment, 359 had not yet completed decision making, and 13 (1.3%) refused decision making assessment. Of the 620, 18 had dementia, 53 had missing data, and 129 had only one valid cognitive assessment. This left 420 persons without dementia with at least two cognitive evaluations for these analyses, including 86 with MCI and 334 without any cognitive impairment.

### Clinical Diagnoses

Based on a previously described structured annual clinical evaluation, all participants were classified by a clinician with respect to Alzheimer’s disease and other conditions known to impair cognitive function (e.g., vascular disease, Lewy bodies) in advanced age according to standard diagnostic criteria, as previously described [11], [12]. The diagnosis of dementia followed the National Institute of Neurologic and Communicative Disorders and Stroke and the Alzheimer’s Disease and Related Disorders Association criteria [1]. The diagnosis of MCI was rendered for individuals who had cognitive impairment but who did not meet criteria for dementia, as previously described [12]. Persons without cognitive impairment were those who did not meet formal criteria for dementia or MCI.

### Assessment of Decision Making

A 12-item version of the Decision Making Competence Assessment Tool was used to examine healthcare and financial decision making [13], [14]. This tool was specifically designed for older adults and uses materials that closely approximate those used in real world settings. The healthcare module involves tables that provide information about HMO plans, and the finance module involves tables that provide financial information about mutual funds. Respondents were asked 6 questions of varying difficulty levels (simple and complex) for each domain (healthcare and financial, respectively); questions assess comprehension and integration of information presented in the tables. Complex problems parallel simple problems but involve more options. For example, one simple finance problem presents information on three mutual funds, including the gross annual return, account management fee, and other features and asks respondents to select the fund with the lowest management fee. Subsequently, a complex problem presents similar information about seven mutual funds and asks respondents to select the most appropriate fund given pre-specified preferences (e.g., Pamela wants a management fee of less than X%, a gross annual rate of return of X%, and a minimum investment of X). The total score is the number of items answered correctly (range = 0–12). This measure has been used in studies of older persons and has adequate psychometric properties [13], [14].

### Assessment of Susceptibility to Scams

Susceptibility to scams was assessed using a 5-item self report measure in which participants rated their agreement using a 7-point likert scale for statements such as “If something sounds too good to be true, it probably is” or “I feel I always have to answer the phone even if I do not know who is calling.” The total score is the average of ratings across the 5 items. The statements were based on findings from the American Association of Retired Persons regarding the personal characteristics of known fraud victims and included items from and similar to those used in the Financial Industry Regulatory Authority Risk Meter, a measure of poor and risky financial decision making that is widely used in finance studies [15]. The intraclass coefficient (ICC) for this measure was 0.63, suggesting moderate internal consistency.

### Assessment of Cognition

Cognition was assessed via a battery of 21 tests, as previously described [11], [12]. Scores on19 tests were used to summarize global cognitive function, including immediate and delayed recall of story A from Logical Memory, immediate and delayed recall of the East Boston Story, Word List Memory, Word List Recall, and Word List Recognition, a 15-item Boston Naming Test, Verbal Fluency, a 15-item reading test, Digit Span Forward, Digit Span Backward, Digit Ordering, Symbol Digit Modalities Test, Number Comparison, two indices from the Stroop Test; a 15-item Judgment of Line Orientation, and a 16-item Standard Progressive Matrices. Global cognition was formed by converting raw scores on the individual tests to z-scores using the mean and standard deviation of the entire cohort and then averaging the z-scores together [17], [18].

## Data Analysis

First, we examined the bivariate associations of decision making and scam with demographics and relevant covariates. Then, to investigate the temporal association between the prior rate of change in cognition and the level of decision making, we first estimated the slope of cognitive decline for each individual. Thus, we fit a general linear mixed model to all available longitudinal cognitive testing data up until the time of decision making, adjusted for baseline age, gender, and years of education. These person-specific slopes were then used in ordinary least squares regressions as the predictor of decision making and susceptibility to scams; all models adjusted for age, sex, education, and starting level of cognition. Programming was done in SAS and models were validated graphically and analytically [16].

## Results

Descriptive data on the overall group (n = 420) are provided in Table 1. The mean decision making total score was 7.2 (SD = 2.9), with higher scores indicating better decision making. The mean scam score was 2.9 (SD = 0.7), with higher scores indicating increased susceptibility. Bivariate correlation analyses revealed that the level of decision making was negatively associated with age (r = −0.26, p<.001) and positively associated with education (r = 0.37, p<.001), MMSE score (r = 0.43, p<.001), global cognition (r = 0.58, p<.001), and male sex (r = 0.16, p<.001). Susceptibility to scams was positively associated with age (r = 0.29, p<.001) and negatively associated with education (r = −0.13, p = .006), MMSE score (r = −0.25, p<.001), and global cognition (r = −0.30, p<.001). Sex was not related to susceptibility to scams (p = 0.970). A higher level of decision making was associated with decreased susceptibility to scams (r = −0.22, p<.001).

**Table 1:** Descriptive data on the overall cohort.

	Overall Group (n = 420)
Age, years (mean, SD)*	83.5 (7.4)
Education, years	15.2 (3.1)
MMSE	28.2 (1.8)
Women, %	319 (76.0)
Non-Hispanic white, %	374 (89.3)
Decision making (total score)	7.2 (2.9)
Financial decision making	3.4 (1.5)
Healthcare decision making	3.9 (1.7)
Scam score	2.9 (0.7)

* Values are mean (SD) or number (percent).

### Decision Making as a Consequence of Cognitive Decline among Persons without Dementia

First, to clarify the temporal relation between cognitive decline and decision making, we conducted analyses examining the relation of the prior rate of change in cognition with the level of decision making among 420 persons without dementia who had completed the decision making assessment and at least 2 prior cognitive evaluations. These analyses allowed us to directly test the hypothesis that impaired decision making is a consequence of cognitive decline in old age. These persons had annual cognitive data for a mean of 5.5 years (SD = 2.8, range: 0.4–13.0). We used the composite measure of global cognition (baseline mean = 0.32, SD = 0.45, range: −1.17–1.40) and constructed a mixed-effects model with a term for time (since baseline in years) to estimate each person’s annual rate of cognitive change (i.e., slope). Cognition declined at a mean of 0.015 unit per year (SD = 0.03; range: −0.20–0.07).

To test the hypothesis that the prior rate of change in global cognition predicted the level of decision making, we constructed a linear regression model with the decision making score as the outcome and terms for global cognitive slope, age at decision making, sex, education, and starting level of cognition. The results of this analysis showed that a more rapid rate of cognitive decline was associated with poorer decision making (p<0.001; Table 2). To clarify this effect, when the rate of decline in global cognition increased by 1 standard deviation, the reduction in the average decision making score was equivalent to the effect of being about 7 additional years older in terms of decision making performance.

**Table 2:** Decision making and scams as a function of cognitive decline*.

	Persons without dementia N = 420		No cognitive impairment only N = 334	
	EST (SE)	P	EST (SE)	P
Decision making				
Age	−0.057 (0.017)	<.001	−0.051 (0.018)	0.005
Female	1.142 (0.276)	<.001	0.949 (0.308)	0.002
Education	0.154 (0.042)	<.001	0.115 (0.047)	0.015
Starting level of cognition	2.284 (0.286)	<.001	2.935 (0.364)	<.001
Cognitive decline	0.229 (0.041)	<.001	0.195 (0.051)	<.001
Susceptibility to scams				
Age	0.019 (0.005)	<.001	0.026 (0.005)	<.001
Female	0.030 (0.080)	0.703	0.062 (0.088)	0.478
Education	−0.019 (0.012)	0.106	−0.017 (0.013)	0.194
Starting level of cognition	−0.152 (0.082)	0.065	−0.195 (0.104)	0.060
Cognitive decline	−0.039 (0.012)	0.001	−0.031 (0.015)	0.037

* Parameter estimates of cognitive decline per 0.01 unit increase in rate of change; estimated from regression models adjusted for age, sex, education, and starting level of cognition.

Because it is possible that factors such as income or race may have influenced the above finding, we repeated the model above after adjustment for these factors. In this analysis, the association of cognitive decline with decision making persisted and was essentially unchanged (estimate for cognitive slope = 0.204, SE = 0.042, p<0.001).

### Relation of Cognitive Decline with Decision Making among Persons without Dementia or Even MCI

Because it is possible that the findings above were driven by the inclusion of persons exhibiting MCI, in the next set of analyses, we excluded those with MCI and repeated the longitudinal analyses to determine whether cognitive decline was associated with poorer decision making among persons without any evidence of cognitive impairment (i.e., no dementia or even MCI). In these analyses restricted to persons with no cognitive impairment (n = 334), the association between cognitive decline and decision making remained robust and was essentially unchanged (Table 2).

### Relation of Cognitive Decline with Susceptibility to Scams among Persons without Dementia

Next, to test the hypothesis that the prior rate of change in global cognitive function predicted the level of susceptibility to scams among persons without dementia, we constructed a linear regression model with the scam score as the outcome and terms for global cognitive slope, age at scam assessment, sex, education, and starting level of cognition. The results of this analysis showed that a more rapid rate of cognitive decline was associated with a greater susceptibility to scams (p<0.001; Table 2). To clarify this effect, when the rate of decline in global cognition increased by 1 standard deviation, the increase in vulnerability to scams was equivalent to the effect of being about 5 years older.

Because it is possible that factors such as income or race may have influenced the above finding, we repeated the model above after adjustment for these factors. In this analysis, the association of cognitive decline with vulnerability to scams persisted (estimate for cognitive slope = −0.038, SE = 0.016, p = 0.004).

### Relation of Cognitive Decline with Scams among Persons without Dementia or MCI

In the final set of analyses, we excluded persons with MCI and repeated the longitudinal analyses to determine whether cognitive decline was associated with susceptibility to scams even among persons without any evidence of cognitive impairment (n = 334). In these analyses, the association between cognitive decline and scams persisted and the magnitude of the effect was essentially unchanged (Table 2).

## Discussion

In a cohort of more than 400 community-based older persons without dementia, we found that a more rapid rate of cognitive decline over about 5.5 years predicted poorer decision making and increased susceptibility to scams among persons without dementia. Further, in analyses restricted to persons without any evidence of cognitive impairment (i.e., no dementia or even MCI at the time of decision making assessment), the relation of the prior rate of cognitive decline with the level of decision making and susceptibility scams persisted. These results suggest that poor decision making and increased susceptibility to scams in old age are consequences of cognitive decline among persons without dementia. Most importantly, even among older persons that most would consider “cognitively healthy”, the very subtle cognitive decline that occurs detrimentally effects decision making and judgment.

It is well recognized that decision making is impaired among persons with dementia, but to date, very little is known about the relation of cognition with decision making among older persons without dementia [9], [17]–[19]. The temporal relations between cognitive decline, decision making and susceptibility to scams among persons without dementia as well as among cognitively intact persons (i.e. no dementia or even MCI at the time of decision making assessment) have major public health implications. There are currently about 40 million persons over age 65 in the United States and by 2030, there will be more than 70 million. Older persons hold the majority of the household wealth and face a myriad of complex and influential financial decisions. Similarly, older persons carry a disproportionate burden of disease and make some of life’s most challenging healthcare decisions (e.g., end of life decisions). Although there is a widespread presumption that decision making is preserved among cognitively intact persons, increasing evidence suggests this is not the case. Experimental data suggest that a sizable proportion of older persons, even those without cognitive impairment, have difficulty making decisions under conditions of uncertainty, and there are numerous examples of older persons making poor financial and healthcare decisions [3]–[5], [20]–[21]. Our findings suggest that many older persons, even those without manifest cognitive dysfunction, may have difficulty making the decisions needed to maintain independence and well-being and may fall prey to scams as a result of very subtle, age-related changes in cognition. If decision making is impaired in many of the 6 million persons currently living with MCI, plus some without any overt cognitive impairment, then poor decision making may threaten the health and well being of literally tens of millions of Americans (without considering the downstream effects on relatives and society at large) and many more worldwide.

Importantly, our findings suggest that impaired decision making may be a very early consequence of cognitive decline, manifest prior to the time a clinical diagnosis of MCI can be made. Although it is now recognized that MCI reflects a process of cognitive decline over some period of time prior to the point at which one meets the threshold for diagnosis of MCI, surprisingly little is known about the consequences of cognitive decline among older persons who do not meet criteria for a diagnosis of cognitive impairment (i.e., MCI or dementia) [2], [12], [22], [23]. Decision making involves complex reasoning, consideration of alternatives and outcomes, patience and time. Our findings suggest that decision making and judgment may be highly vulnerable to the very subtle cognitive changes that occur with what most consider “healthy cognitive aging” and are widely considered benign. The present findings challenge the view that subtle, age-related changes in cognition are benign and instead suggest that these changes have deleterious effects on decision making, a behavior that is essential for functioning successfully in the world. Moreover, these findings suggest that, just as MCI is often the precursor to dementia, poor decision making may be a harbinger of the future development of MCI or dementia. Thus, decision making warrants further investigation as a potentially important and early indicator of adverse health outcomes in old age.

Although the neurobiologic basis of the association of cognition with decision making remains unknown in old age, it is now well known that Alzheimer disease pathology, cerebrovascular disease, and other neuropathologies are commonly found in the brains of older persons without dementia or MCI, and Alzheimer pathology is associated with subtle decrements in episodic memory among persons without overt cognitive impairment [24], [25]. Given that common neuropathologies are related to age-related cognitive decline, we suspect that these pathologies may also impair the complex thinking processes that underlie decision making. Future studies are needed to elucidate the neurobiologic basis of the association of cognition with decision making, but the available data on AD pathology in non-demented persons raises the possibility that impaired decision making in older persons is not simply due to aging; rather, it is the result of subclinical disease.

The strengths and limitations of this study should be noted. One novel feature of this study was that it employed a measure of decision making that very closely approximates the complex financial and healthcare decisions older persons make in real world settings. Another strength is that it focused on non-demented, community-based persons, including some with clinically diagnosed MCI and a large number of persons without manifest cognitive impairment at the time of the decision making assessment. Participants were evaluated over an average of 5 years with a very high rate of follow-up participation and multiple dimensions of cognition and decision making were assessed with previously established, psychometrically sound measures. These factors enhanced our ability to characterize person-specific changes in cognition. The main limitation is that participants were selected; thus, it is important to replicate these findings in other groups. In addition, MCI (like dementia) reflects the result of a process of cognitive decline over a period of time prior to the point at which one meets criteria for MCI, and it is possible that some persons included in the group of non-impaireds (e.g., no MCI or dementia) may have been on their way to developing MCI or perhaps dementia even though they did not meet clinical criteria at the time of the decision making assessment. Finally, a limitation was the specific focus on cognition; personality and other factors also influence decision making, and future research is greatly needed to clarify the determinants of age-related changes in decision making. This line of research has the potential to reduce the problem posed by the exploitation of vulnerable older persons and may facilitate interventions to improve decision making. Ultimately, such interventions could promote independence, health, and well-being among millions of older persons.
